# Restraint use and injury in forward and rear-facing infants and toddlers involved in a fatal motor vehicle crash on a U. S. Roadway

**DOI:** 10.1186/s40621-019-0200-4

**Published:** 2019-05-29

**Authors:** Yu-Yun Huang, Chang Liu, Joyce C. Pressley

**Affiliations:** 1 Columbia University Mailman School of Public Health Departments of Epidemiology and Health Policy and Management, 722 West 168th St, New York, NY 10032 USA; 2 Health Policy and Management, New York, NY USA; 3 Center for Injury Epidemiology and Prevention at Columbia, New York, NY USA

**Keywords:** Motor vehicle injury, Infant, Toddlers, Child safety seats, Rear-facing, AAP guidelines

## Abstract

**Background:**

Use of appropriate child passenger safety restraints reduces injury in infants, with rear facing restraints favored over forward facing. In 2011, the American Academy of Pediatrics (AAP) began recommending that infants and children under the age of 2 years be restrained in a rear-facing seat installed in the vehicle’s rear seat. This study examines the practice of rear-facing restraints pre- and post-AAP recommendations for children under 2 years.

**Methods:**

Data from the Fatality Analysis Reporting System (FARS) from 2008 to 2015 were used to examine restraint status and injuries in rear-seated infants and toddlers aged 0 to less than 2 years involved in fatal collisions (*n* = 4966). Subpopulation analyses were conducted on 1557 children with seat facing direction recorded. Multivariable logistic regression was used to generate odds ratios (OR) with 95% confidence intervals (CI). Covariates considered for inclusion in the multivariable model included passenger characteristics (age, gender, seating position), driver characteristics (age, gender, seat belt status, alcohol status, drug status, previous traffic violations), vehicle characteristics (vehicle type), and crash-level characteristics (day/night, weekday/weekend, rush hour, expressway/surface street, urban/rural).

**Results:**

Approximately 6.7% (330 of 4996) of infants and toddlers were unrestrained with mortality that was approximately triple that of restrained infants (40.0% vs 13.7%, *P* < 0.0001). In multivariable adjusted models, predictors of an infant being unrestrained included unrestrained driver (OR: 3.17, 95% CI: 2.38–4.21), driver aged less than 20 years (OR: 2.18, 95% CI: 1.42–3.34), driver alcohol use (OR: 2.21, 95% CI: 1.42–3.44), center-seated infant (OR: 1.55, 95% CI: 1.19–2.03) and weekday crash (OR: 1.52, 95% CI: 1.12–2.01). Of all rear-seated children whose restraint status were reported (4966), rear-facing restraint use increased from 5.0% to 23.2% between 2008 and 2015 (*P* < 0.0001). The odds of rear-facing restraint use increased after introduction of the AAP guideline among infants aged 0 to < 1 year old (OR: 2.12, 95% CI: 1.46–3.10) and among toddlers aged 1 to < 2 years old (OR: 1.97, 95% CI: 1.03–3.79).

**Conclusion:**

Trends in the use of rear-facing child restraints improved over the timeframe of this study, but remain low despite the introduction of AAP guidelines and the strengthening of child restraint laws.

## Background

Previous studies have reported that child restraints are effective at lowering mortality and lessening injury severity (Agran et al., 1998; Elliott et al., 2006; Hertz, 1996; Sauber-Schatz et al., 2014; Huang et al., 2016). It is estimated that nearly three-quarters (71%) of infants in passenger cars and 58% of infants in light trucks and vans who die without a child safety seat would have survived had they been restrained properly in a child safety seat (Hertz, 1996). Proper restraint use is influenced by several factors including age, height and weight of the child.

The American Academy of Pediatrics (AAP) recommendation that infants and toddlers aged 0–2 years be in a rear-facing child restraint in the vehicle’s rear seat, originally introduced in 2011, has been updated recently (Durbin et al., 2018) to a policy that now recommends children remain rear-facing for as long as allowable by the car seat manufacturer’s guidelines. The National Highway Traffic Safety Administration (NHTSA) suggests that infants < 1 year of age ride in the vehicle’s rear seat in a rear-facing car seat, and for children over the age of 1 year, that they remain seated similarly for as long as height and weight permit (NHTSA, 2011). Currently, several states have laws or regulations that require infants less than 1 year of age or < 20 lbs. to be transported rear-facing in the vehicle’s rear seat with other states requiring children younger than age 2 or < 40 pounds ride rear-facing in the rear seat (GHSA, 2017).

This study examined trends in infant restraint seat direction in the United States (U.S.). Observed before and after the 2011 policy recommending that infants aged less than two years ride in a rear-facing restraint. Specifically, it examines occupant mortality in infants aged 0 to < 1 and 1 to < 2 years of age who were rear-seated: (1) to characterize predictors of restraint use and non-use; (2) to describe the characteristics and compare trends for rear-facing restraint use pre- and post-2011 AAP recommendations; and (3) to describe the characteristics of fatal crashes with missing restraint documentation.

## Methods

### Data source

Fatality Analysis Reporting System (FARS) data from 2008 to 2015 were obtained from the National Highway Traffic Safety Administration’s (NHTSA) public use data files (NHTSA, 2015). FARS is a census of all crashes on U.S. public roads in which at least one person died within 30 days of the crash. FARS contains person-, vehicle- and crash-level variables including driver and passenger characteristics, drug and alcohol information, restraint use, seating position, severity of injury, traffic violations, vehicle body type, crash time and other information. FARS is a publicly available de-identified data set, and this study was deemed exempt by the Columbia University Institutional Review Board (IRB).

### Study population with descriptive information on exclusions

**Fig. 1:**
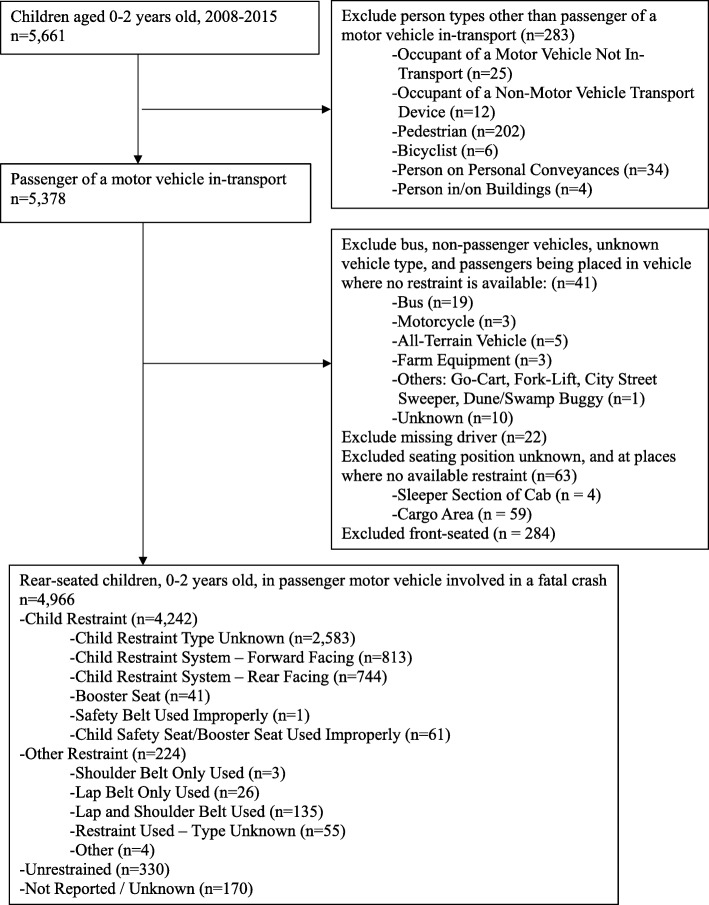
Study population flow diagram, Fatality Analysis Reporting System, 2008–2015

Of the 5661 children aged 0 to less than 2 years old involved in a fatal collision on a U.S. roadway, 5378 (95.0%) were occupants in passenger vehicles (Fig. 1). Infants were excluded who were: (1) not a passenger in a motor vehicle in transport (e.g. pedestrians) (*n* = 283, 5.0%); (2) not being transported in a passenger vehicle equipped with safety belts (*n* = 41, 0.72%); (3) missing driver information (*n* = 22, 0.39%); (4) seated in vehicle regions where no restraint was available (sleeper section of cab or enclosed/unenclosed cargo area) (*n* = 63, 1.11%); or (5) who were seated in the vehicle’s front seat (*n* = 284, 5.0%).

The final study population included 4966 (87.7%) infants and toddlers who were seated in the vehicle’s rear seat (Fig. 1). More than half (63.3%) of the records for 4242 restrained children did not specify whether they were in a forward-facing or rear-facing restraint system. A subpopulation analysis examined 1557 rear-seated children who had data indicating the direction the child restraint was facing.

### Variable classification

#### Outcomes

##### Child Restraint

Child restraint status was examined as follows: (1) a dichotomous seat-variable for restrained vs. unrestrained; (2) a dichotomous seat-direction variable (rear-facing or forward-facing); and as (3) a four-category variable of child restraint, other restraint, unrestrained, and unknown restraint.

##### Child injury severity

Child injury severity was categorized as not injured, injured (including non-incapacitating injury, incapacitating injury, injured but severity unknown), died within 30 days of crash, or unknown.

#### Exposures

##### Pre- and post- American Academy of Pediatrics guideline

In late March 2011, the American Academy of Pediatrics (AAP) published a policy recommending that all infants and toddlers ride in a rear-facing seat until 2 years of age (Committee on Injury Violence and Prevention, 2011). Crashes that occurred before April 1, 2011 were defined as Timeframe 1 (pre-AAP policy) and crashes occurring afterward as Timeframe 2 (post-AAP policy). The AAP updated this recommendation in August 2018 to remove the age specification and to say that most children could remain rear-facing beyond their second birthday, but this clarification occurred after our study period (Durbin et al., 2018).

#### Person-level characteristics

##### Child passenger age and gender

Child passenger age was examined as a dichotomous variable with age groups 0 to less than 1, and 1 to less than 2 years. Gender was categorized as male, female, and unknown.

##### Child seating position

Infants and toddlers in row 2 or higher were categorized as rear-seated. Children being transported in row 1 of a vehicle were categorized as front-seated. Child seating position was further categorized as center-seated or seated outboard.

##### Driver age and gender

Driver age groups were < 20, 20–29, 30–64, ≥65 years, and unknown. Gender was categorized as male, female, or unknown.

##### Driver Restraint

Driver’s restraint status was categorized as restrained (shoulder belt only, lap belt only, shoulder and lap belt, restraint used with unknown type, safety belt used improperly, or other restraint), unrestrained, or unknown.

##### Driver alcohol and drug use

Driver’s alcohol status was categorized as positive, negative or unknown. The driver was considered alcohol positive if police reported alcohol or if the driver had a blood alcohol concentration (BAC) ≥ 0.01. Of the 294 drivers categorized as positive BAC, 77.6% of drivers had BAC ≥ 0.08. Driver’s drug use was categorized as positive, negative or unknown (either not tested, or tested but result unknown). A driver was considered positive for drugs if the police reported drug involvement or if the driver tested positive for drugs.

##### Driver’s previous moving violations

Previous violations included having a history in the last three years of driver license suspensions or revocations for a moving violation, driving while intoxicated, speeding, or other moving violations within three years of the crash date.

#### Vehicle-level characteristics

##### Vehicle body type

Vehicles were categorized as passenger cars, SUVs, vans, pickups or other.

Crash-Level Characteristics.

##### Weekday/Weekend

Weekend was defined as 6:00 PM Friday to 6:00 PM Sunday. Weekday and weekend were analyzed as a dichotomous variable.

##### Day/Night

Daytime was defined as 6:00 AM to 5:59 PM and nighttime was defined as 6:00 PM to 5:59 AM.

##### Weekday rush hour

Weekday rush hour was defined as 7:00 AM to 9:30 AM or 3:30 PM to 5:59 PM.

##### Expressways/Surface streets

Expressways were defined as roadways with limited access, while “surface streets” comprised all other roadways.

##### Urban/Rural

The trafficway on which the crash occurred was classified as urban, rural or unknown.

### Statistical analysis

The Chi-square (χ2) test was used in analyses of associations between child restraint status and injury severity and potential covariates. Significance was defined as a *P*-value < 0.05. Unadjusted and adjusted odds ratios (OR) with 95% confidence intervals (CI) for child restraint use, car seat direction, missing child restraint documentation, and injury/mortality were analyzed using univariable and multivariable logistic regression. Except for age and gender, variables that were not significant predictors of the outcome were not included in the final models of the adjusted association between each predictor and the outcome. Subpopulation analyses were performed on 4466 children who were restrained (in any type of restraint) and on 1557 rear-seated child passengers whose child restraint direction was recorded. All analyses were conducted using SAS version 9.4 (SAS Institute Inc, 2014, Cary, North Carolina).

## Results

### Study population

Of the 5661 children aged 0 to less than 2 years old involved in a fatal motor vehicle collision during the study timeframe, 4966 were rear-seated and comprised the population for this study (Fig. 1).

### Restraint status in rear-seated children

Among 4966 rear-seated passengers, 4242 (85.4%) were restrained in a child restraint system, 224 (4.5%) were restrained in a non-child restraint system, 330 (6.7%) were unrestrained and 170 (3.4%) had unknown restraint status (Table 1). Drivers of unrestrained children were nearly 2.4 times more frequently unrestrained compared to drivers of children restrained in a child restraint (*P* < 0.0001)(Table 1).

**Table 1:** Child restraint use among infants aged 0 to < 2 years involved in fatal crashes, FARS 2008–2015

Variables	Child Restraint ^a^ n (%)	Other Restraint ^a^ n (%)	Unrestrained n (%)	Unknown n (%)	Total	Chi-square χ^2^(*p* value) ^b^
Total (n, row%)	4242 (85.4)	224 (4.5)	330 (6.7)	170 (3.4)	4966	
Passenger characteristics						
Passenger age (years)						1.9 (0.60)
0 to < 1	1857 (43.8)	88 (39.3)	145 (43.9)	72 (42.4)	2162 (43.5)	
1 to < 2	2385 (56.2)	136(60.7)	185 (56.1)	98 (57.6)	2804 (56.5)	
Passenger gender						2.3 (0.50)
Male	2210 (52.2)	117 (52.2)	169 (51.2)	98 (58.0)	2594 (52.4)	
Female	2021 (47.8)	107 (47.8)	161 (48.8)	71 (42.0)	2360 (47.6)	
Seating position						11.1 (0.011)
Center-seated	1221 (29.4)	60 (27.4)	104 (38.7)	35 (30.2)	1420 (29.8)	
Outboard-seated	2936 (70.6)	159 (72.6)	165 (61.3)	81 (69.8)	3341 (70.1)	
Injury severity						229.8 (< 0.0001)
Not injured	1687 (39.8)	94 (42.0)	27 (8.2)	52 (30.6)	1860 (37.4)	
Injured	1124 (26.5)	61 (27.2)	122 (37.0)	47 (27.6)	1354 (27.3)	
Died	583 (13.7)	31 (13.8)	132 (40.0)	42 (24.7)	788 (15.9)	
Unknown	848 (20.0)	38 (17.0)	49 (14.9)	29 (17.1)	964 (19.4)	
Driver characteristics						
Driver age (years)						41.4 (< 0.0001)
< 20	270 (6.4)	12 (5.4)	35 (10.6)	NR	324 (6.5)	
20 to 29	2149 (50.7)	98 (43.8)	148 (44.8)	73 (42.9)	2468 (49.7)	
30 to 64	1746 (41.2)	113 (50.5)	133 (40.3)	90 (53.0)	2082 (41.9)	
> =65	77 (1.8)	NR	14 (4.2)	NR	92 (1.9)	
Driver gender						12.7 (0.0054)
Male	1697 (40.0)	102 (45.5)	156 (47.3)	82 (48.2)	2037 (41.0)	
Female	2546 (60.0)	122 (54.5)	174 (52.7)	88 (51.8)	2929 (59.0)	
Driver restraint status						113.8 (< 0.0001)
Restrained	3387 (83.5)	197 (90.4)	183 (60.8)	80 (73.4)	3847 (82.0)	
Unrestrained	671 (16.5)	21 (9.6)	118 (39.2)	29 (26.6)	839 (17.9)	
Driver’s alcohol status						34.0 (< 0.0001)
Positive	225 (5.3)	17 (7.6)	46 (13.9)	14 (8.2)	302 (6.1)	
Negative	1367 (32.2)	52 (23.2)	105 (31.8)	44 (25.9)	1568 (31.6)	
Unknown	2650 (62.5)	155 (69.2)	179 (54.2)	112 (65.9)	3096 (62.3)	
Driver’s drug status						5.5 (0.14)
Positive	429 (10.1)	13 (5.8)	42 (12.7)	18 (10.6)	502 (10.1)	
Negative	2253 (53.1)	104 (46.4)	160 (48.5)	91 (53.5)	2608 (52.5)	
Unknown	1560 (36.8)	107 (47.8)	128 (38.8)	61 (35.9)	1856 (37.4)	
Previous violations						13.7 (0.0033)
Yes	1448 (34.8)	79 (35.3)	125 (37.9)	77 (47.2)	1729 (35.6)	
No	2717 (65.2)	135 (60.3)	187 (56.7)	86 (52.8)	3124 (64.4)	
Vehicle characteristics						
Vehicle type						11.7 (0.46)
Passenger car	1999 (47.1)	101 (45.3)	133 (40.4)	72 (42.35)	2305 (46.5)	
SUV	1369 (32.2)	66 (29.6)	118 (35.8)	55 (32.4)	1608 (32.5)	
Van	480 (11.3)	34 (15.3)	41 (12.5)	22 (12.9)	577 (11.6)	
Pickup truck	384 (9.1)	22 (9.9)	37 (11.3)	19 (11.2)	462 (9.3)	
Other	NR	NR	NR	NR	4 (0.1)	
Crash-level characteristics						
Day/Night						17.9 (0.0005)
Day	2711 (64.0)	137 (61.2)	178 (53.9)	93 (55.4)	3119 (62.9)	
Night	1524 (36.0)	86 (38.4)	152 (46.1)	75 (44.6)	1837 (37.1)	
Weekday/Weekend						12.6 (0.0057)
Weekday	2765 (65.3)	143 (63.8)	235 (71.2)	93 (55.4)	3241 (65.3)	
Weekend	1470 (34.7)	81 (36.2)	95 (28.8)	75 (44.6)	1725 (34.7)	
Weekday rush hour						4.5 (0.21)
Yes	1188 (28.1)	51 (22.9)	81 (24.6)	45 (26.8)	1365 (2.5)	
No	3047 (72.0)	172 (77.1)	249 (75.5)	123 (73.2)	3591 (72.5)	
Roadway type						11.4 (0.0097)
Expressway	2833 (69.4)	127 (59.4)	215 (68.3)	104 (63.8)	3278 (68.7)	
Surface street	1250 (30.6)	87 (40.7)	100 (31.8)	59 (36.2)	1496 (31.3)	
Urban/Rural						19.2 (0.0003)
Urban	1597 (43.8)	102 (53.1)	106 (37.2)	75 (55.6)	1880 (44.2)	
Rural	2049 (56.2)	90 (46.9)	179 (62.8)	60 (44.4)	2378 (55.9)	

^a^Child restraint include booster seat, rear-facing/forward-facing car seat, and child restraint type unknown. Other restraint includes lap belt, shoulder belt, and helmet

^b^Unknown categories were not used in the calculation of the chi-squares; *NR* not reported due to small numbers

### Injury/mortality in unrestrained rear-seated infants and toddlers

Among children aged 0 to less than 2 years old involved in a fatal crash, mortality was nearly triple in unrestrained passengers compared to restrained passengers (40.0% vs 13.7%, *p* < 0.0001) (Table 1).

### Factors associated with being unrestrained

Factors associated with the transport of an unrestrained infant passenger are shown in Table 2. In multivariable models, independent predictors of an infant being unrestrained included driver age (drivers aged < 20 years compared with drivers aged 20 to 29), driver restraint status (unrestrained), driver alcohol status (yes-alcohol positive), passenger seating position (center-seated compared to seated outboard), crash time (weekday compared to weekend) (Table 2). There was a tendency for infants traveling at night to be unrestrained in the unadjusted model. In both unadjusted and adjusted multivariable models, driver’s age < 20 years and driver’s unrestrained were predictive of having an unrestrained infant passenger (Table 2). Drivers who tested positive or were police reported as alcohol-involved crash were more likely to have an unrestrained infant compared to those who were alcohol negative (adjusted OR: 2.21, 95% CI: 1.42–3.44). In unadjusted models, the odds of male drivers transporting an infant unrestrained were 33% higher compared to female drivers but this effect was not significant in the adjusted models. Center-seated infants compared to those seated outboard and weekday compared to weekend crashes were associated with infant passengers being unrestrained in both unadjusted and adjusted models (Table 2).

**Table 2:** Predictors of unrestrained infants aged 0 to < 2 years involved in fatal crashes, FARS 2008–2015

Variables	Unadjusted, unrestrained OR (95% CI)	Multivariable adjusted, unrestrained OR (95% CI)
Passenger characteristics		
Passenger age (years)		
0 to < 1	Ref	
1 to < 2	0.98 (0.79–1.23)	
Passenger gender		
Male	Ref	
Female	1.04 (0.83–1.30)	
Seating position		
Center seated	1.52 (1.18–1.96)	1.55 (1.19–2.03)
Seated outboard	Ref	Ref
Driver characteristics		
Driver age (years)		
< 20	1.97 (1.35–2.88)	2.18 (1.42–3.34)
20 to 29	Ref	Ref
30 to 64	1.09 (0.85–1.38)	1.16 (0.87–1.54)
≥ 65	2.43 (1.29–4.57)	2.00 (0.88–4.54)
Driver gender		
Male	1.33 (1.06–1.66)	
Female	Ref	
Driver restraint status		
Restrained	Ref	Ref
Unrestrained	3.34 (2.61–4.27)	3.17 (2.38–4.21)
Driver’s alcohol status		
No	Ref	Ref
Yes	2.57 (1.77–3.73)	2.21 (1.42–3.44)
Unknown	0.86 (0.67–1.11)	1.03 (0.77–1.39)
Driver’s drug status		
No	Ref	
Yes	1.40 (0.98–2.00)	
Unknown	1.13 (0.89–1.44)	
Previous violations		
No	Ref	
Yes	1.25 (0.99–1.58)	
Vehicle characteristics		
Vehicle type		
Passenger car	Ref	
SUV	1.30 (1.00–1.68)	
Van	1.44 (0.99–2.10)	
Pickup truck	1.26 (0.88–1.81)	
Crash-level characteristics		
Day/Night		
Day	Ref	
Night	1.51 (1.21–1.89)	
Weekday/Weekend		
Weekday	1.32 (1.03–1.69)	1.52 (1.12–2.01)
Weekend	Ref	Ref
Weekday rush hour		
Yes	Ref	
No	1.18 (0.91–1.53)	
Roadway type		
Expressway	Ref	
Surface Street	1.03 (0.81–1.32)	
Urban/Rural		
Urban	Ref	
Rural	1.34 (1.05–1.72)	
Unknown	1.15 (0.80–1.65)	

### Trends in rear-facing Restraint use

**Fig. 2:**
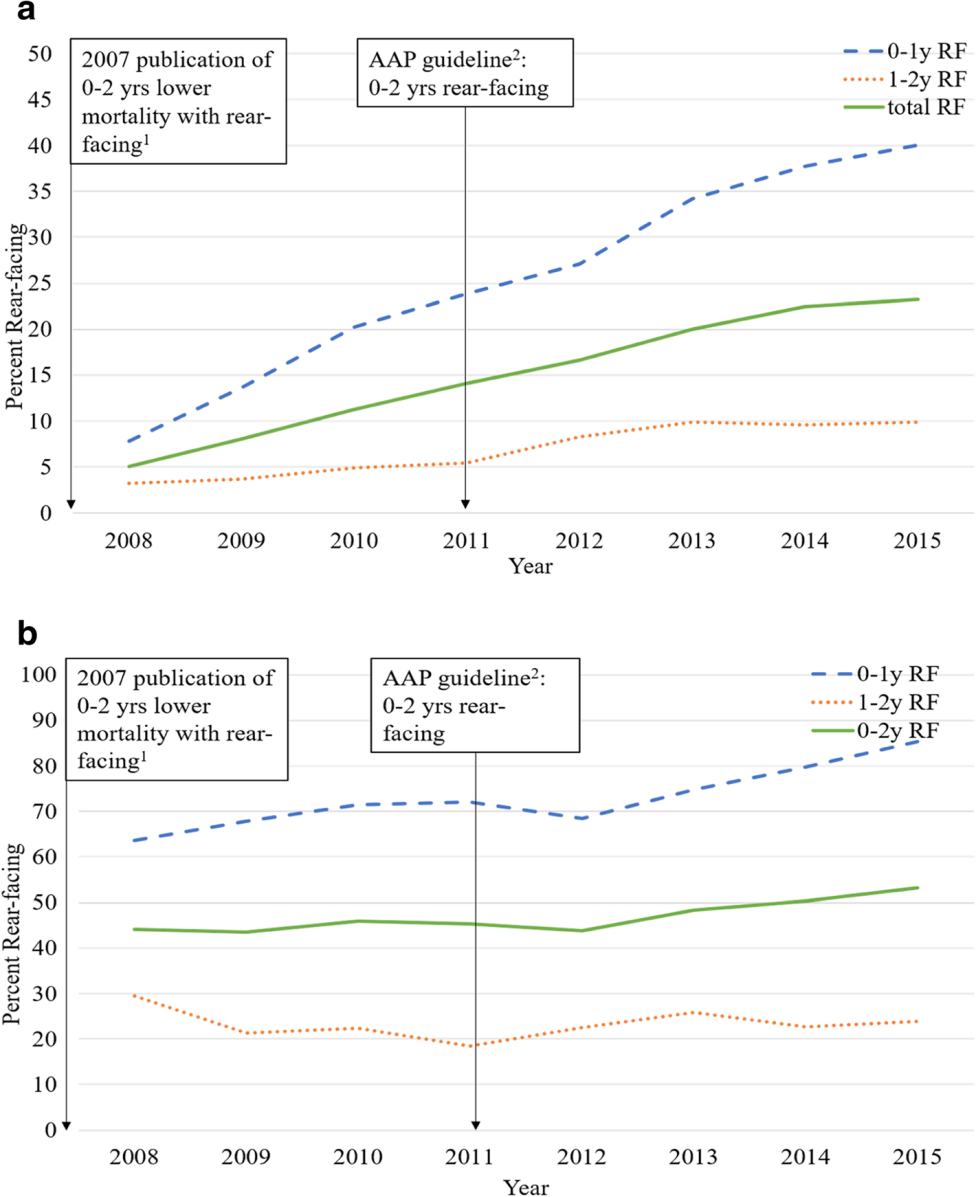
**a** Trends in Annual Percent of Rear-Seated Rear-Facing Infants and Toddlers. **a**. Trends in annual percent of rear-seated, rear-facing infants shown by a dashed line before and after release of the AAP guideline with toddlers aged 1-2 years shown in the dotted line. Total for infants and toddlers is shown in the solid line, FARS 2008–2015 (*n* = 4996). **b**. Trends in Annual Percent of Rear-Seated Rear-Facing Infants and Toddlers among Children within the Same Age Group. **b**.Trends in annual percent of rear-seated infants (shown by dashed line) and toddlers (shown by dotted line) who were rear-facing, FARS 2008–2015 (n = 1557). ^1^Henary B, Sherwood CP, Crandall JR, et al. Car safety seats for children: rear facing for best protection. Injury Prevention. 2007;13(6):398-402. ^2^AAP recommended infants and toddlers ride rear facing until 2 years of age in March 2011

While child restraint use in crashes involving a fatality was stable at approximately 85% across the study period, the transport of all infants and toddlers aged 0 to less than 2 years old for whom restraint status was reported and who were being transported in a rear-facing restraint system increased from 5.0% to 23.2% from 2008 to 2015 (*P* < 0.0001) (Fig. 2a). During the pre-AAP policy era, the odds of being transported in a rear-facing restraint increased for infants aged 0 to < 1 year old (OR: 3.62, 95% CI: 1.61–8.10). However, this increase was not significant for toddlers aged 1 to < 2 years old (OR: 1.96, 95% CI: 0.62–6.21). During the post-AAP policy era, the odds of an infant aged 0 to < 2 years old being transported in a rear-facing restraint increased (OR: 1.81, 95% CI: 1.33–2.47). Similar to pre-AAP policy era, the increase was higher among children aged 0 to < 1 year old (OR: 2.12, 95% CI: 1.46–3.10) compared to children aged 1 to < 2 years old (OR: 1.97, 95% CI: 1.03–3.79).

Among 4242 children who were restrained in a child restraint system, the majority were missing data on whether the child was in a forward or rear facing restraint direction. The proportion of children with missing data on forward or rear-facing restraint direction decreased significantly from 88.6% in 2008 to 56.3% in 2015 (χ2 = 178.8, *p* < 0.0001). Among the 1557 children aged 0 to < 2 years with restraint direction recorded, the proportion who were rear-facing increased during post-AAP policy era from 48.6% to 53.1%. Children aged 0 to < 1 years old increased from 73.7% to 85.4% and children age 1 to < 2 years improved from a base of 19.7% to 23.9%) (Fig. 2b).

### Factors associated with rear-facing Restraint use

In the subpopulation of 1557, infant passengers aged 0 to < 2 years whose restraint direction was reported, 74.6% were compliant with the NHTSA recommendation that all infants 0 to < 1 year of age travel in rear-facing car seats; 47.8% were compliant with the AAP guideline that all infants 0 to < 2 year of age travel in rear-facing car seats. Infants aged 0 to < 1 year old were more likely to be rear-facing when compared to 1 to < 2 years old (OR: 10.04, 95% CI: 7.95–12.67).

Among infants with restraint direction reported, center-seated infants were more likely to be rear-facing compared to infants seated in an outboard position in multivariable analysis (adjusted OR: 1.41, 95% CI: 1.13–1.75). In multivariable analysis infant passengers who traveled in SUVs were more likely to be rear-facing than infants traveling in cars (adjusted OR: 1.31, 95% CI: 1.05–1.65).

### Factors associated with missing/unknown Restraint facing direction

The restraint facing direction was more likely to be reported for infants than for toddlers (44.6% vs 40.3%, *p* = 0.004) as were injured children compared to uninjured ones (44.4% vs 38.8%, *p* = 0.0002). Restraint type was more likely to be reported when crashes occurred on expressway than on surface street (42.1% vs 37.8, *p* = 0.007) (Not shown).

### Mortality in front-seated infants and toddlers

There were 284 front-seated child passengers who were excluded from the analysis of rear-seated infants and toddlers aged 0 to < 2 years. Among 912 fatally-injured infants aged 0 to < 2 years, 812 were rear-seated and previously reported. Of the 100 (10.9%) who were front-seated and excluded from previous analysis, 64.0% were unrestrained. Mortality was higher in front-seated compared to rear-seated children (19.6% vs. 13.7%, *p* = 0.038). The proportion of infants in pickup trucks who were front-seated was approximately five times that of other passenger vehicles (19.6% vs 3.5%; *P* < 0.0001).

## Discussion

This analysis of all fatal crashes occurring on a U.S. roadway from 2008 to 2015 was conducted to evaluate trends in the use of rear-facing child restraints pre- and post-release of an AAP guideline recommending this policy. Following release of the AAP guideline for rear facing seat restraint for infants and toddlers younger than 2 years of age, the proportion of infants being transported in a rear-facing restraint system increased among children involved in a fatal motor vehicle crash. Although this increase was much larger for infants aged 0 to < 1 year of age, with an increase that was approximately five-fold higher in 2015 compared to the baseline year of 2008, rear-facing restraint use remained unacceptably low. The analysis was first attempted for all rear-seated children aged less than two years of age to assess restraint use and restraint direction. However, due to the large quantity of unreported and missing restraint direction data, a subpopulation analysis was conducted in children with restraint direction recorded.

While the majority of children were restrained in a child restraint system, about one-fifth of children were unrestrained or restrained in a non-child restraint system. The finding that unrestrained children involved in a fatal crash were more likely to be transported by drivers who were unrestrained, younger (aged < 20 years) and positive for alcohol is consistent with previous studies (Hertz, 1996; Huang et al., 2016; Oh et al., 2017). These findings of lack of restraint use by drivers suggests an area where enforcement of seat belt laws in adults transporting children might improve the safety of child passengers as well.

An early study which used the National Automotive Sampling System Crashworthiness Data System (NASS-CDS) database found that infants in rear-facing restraints had lower mortality and 75% fewer serious injuries (including death) compared to those in forward-facing seats (Henary et al., 2007). In 2017, reanalysis of this data with a slightly extended timeframe and survey-weighted Chi-Square tests was conducted on the sampled data. This updated study found that both infants and toddlers 0–11 months old and 12–23 months old tended to experience fewer injuries in rear-facing than front-facing restraints, but the findings failed to reach statistical significance (McMurry et al., 2018). Subsequent biomechanical work on forward- and rear-facing seats has been conducted. Recently, the AAP strengthened their recommendation that infants and very young children ride rear-facing for as long as feasible, which may be beyond their second birthday (Durbin et al., 2018).

Several car seat manufacturers have developed rear-facing car seats for children older than 1 year that accommodate larger height and weight measurements than the originally introduced seats. Despite AAP and NHTSA recommendations and the availability of seats to accommodate rear-facing infants and toddlers, some studies report that up to three-quarters are transported forward-facing earlier than recommended (Macy et al., 2015; NHTSA, 2009; NHTSA, 2016; O'Neil et al., 2011; Winston et al., 2004; Arbogast et al., 2002). Our finding that children in larger SUVs were more likely to be restrained in a rear-facing restraint than those in smaller cars suggests the need for further investigation as to the role that vehicle size and child restraint design might play in early forward-facing restraint direction.

Parental sources of information regarding when to transition to forward-facing car seats are most frequently obtained from car seat packaging and clinicians (doctor/nurse) (Macy et al., 2015). Parents who received information from car seat sellers were more likely to turn their child’s car seat to face forward at 1 year old, while parents who had knowledge of AAP guidelines were less likely to turn car seat forward-facing before age 1. Although 69% of the parents had heard of AAP guidelines, there is still improvement for clinicians to educate parents on current recommendations for infant and toddler car seat safety (Macy et al., 2015). Education that emphasized the benefits of rear-facing restraints was reported to promote the intent and attitude for following rear-facing recommendations.

Improvements in legislation has been promoted as having potential to increase the use of rear-facing restraints. This is thought to establish community safety norms (Macy et al., 2015). However, there has been a time lag in knowledge diffusion and policy adoption. Even though the AAP published rear-facing guidelines in 2011, the first state law adoption did not occur until 2015. As of 2018, only eight states explicitly required the use of a rear-facing car seat until age 2. The uneven legislation among states suggests an opportunity for improved communication among public health researchers, advocates, concerned citizen groups and legislators to promote more effective policymaking (Bruce et al., 2011; Bae et al., 2014).

This study has limitations. Due to the large amount of unreported and missing restraint direction data, it is unknown how children with missing reported seat-facing position may differ from children whose rear-facing position is known. Furthermore, FARS includes only infants and toddlers involved in fatal crashes. Because having a child unrestrained or improperly restrained may have contributed to an increased likelihood to be fatally injured, the results of this study may not be generalizable to the total population of all passengers in this age range. During and since this study data were collected, several states have passed laws to require rear-facing restraints. Further study is needed to examine ways to improve the impact of changing laws on restraint direction.

More than two-thirds of infants and toddlers did not have data on restraint direction recorded. This suggests the need to conduct educational programs to encourage and train law enforcement personnel on the importance of recording restraint use and restraint direction. Improved data collection could facilitate improved examination of factors associated with a rear-facing seating position compared to forward-facing restraints in children involved in fatal crashes and with more in-depth examination of crash and vehicle factors associated with injury outcomes.

## Conclusions

In summary, trends in rear-facing restraint use improved over the timeframe of this study. The majority of toddlers aged 1 to < 2 years who are involved in a fatal motor vehicle collision with documented restraint direction are not rear-facing. Despite AAP guidelines and the strengthening of state child restraint laws, an unacceptably low proportion of infants and toddlers are being transported in accordance with current best practices.

## Abbreviations

AAP: American Academy of Pediatrics
BAC: Blood Alcohol Concentration
CI: Confidence Interval
FARS: Fatality Analysis Reporting System
NHTSA: National Highway Traffic Safety Administration
SUV: Sport utility vehicles

## References

[CR1] Agran PF, Anderson CL, Winn DG (1998). Factors associated with restraint use of children in fatal crashes. Pediatrics.

[CR2] Arbogast KB, Durbin DR, Kallan MJ, Menon RA, Lincoln AE, Winston FK (2002). The role of restraint and seat position in pediatric facial fractures. J Trauma Acute Care Surg.

[CR3] Bae, JY, et al., Child passenger safety laws in the United States, 1978–2010: Policy diffusion in the absence of strong federal intervention. 2014. 100: p. 30–37. 10.1016/j.socscimed.2013.10.035 PMC3899584 24444836

[CR4] Bruce, BS, et al., Predicting parents’ use of booster seats. 2011: p ip 2010.029181.22. 10.1136/ip.2010.029181 21415070

[CR5] Committee on Injury Violence and Prevention (2011). Child passenger safety. Pediatrics.

[CR6] Durbin DR, Hoffman BD, the Council on Injury (2018). Violence and Poison Prevention. Child Passenger Safety. Pediatrics.

[CR7] Elliott MR, Kallan MJ, Durbin DR, Winston FK (2006). Effectiveness of child safety seats vs seat belts in reducing risk for death in children in passenger vehicle crashes. Archives of Pediatrics & Adolescent Medicine.

[CR8] GHSA. State Laws: Child Passenger Safety. http://www.ghsa.org/state-laws/issues/Child-Passenger-Safety. Accessed 18 May 2017.

[CR9] Henary B, Sherwood CP, Crandall JR (2007). Car safety seats for children: rear facing for best protection. Injury Prevention.

[CR10] Hertz E. Revised estimates of child restraint effectiveness. NHTSA Research Note; 1996. https://crashstats.nhtsa.dot.gov/Api/Public/ViewPublication/96855. Accessed 18 May 2017.

[CR11] Huang Y., Liu C., Pressley J. C. (2016). Child Restraint Use and Driver Screening in Fatal Crashes Involving Drugs and Alcohol. PEDIATRICS.

[CR12] Macy ML, Butchart AT, Singer DC, Gebremariam A, Clark SJ, Davis MM (2015). Looking back on rear-facing car seats: surveying U.S. parents in 2011 and 2013. Acad Pediatr.

[CR13] McMurry TL, Arbogast KB, Sherwood CP, Vaca F, Bull M, Crandall JR and Kent RW. Rear-facing versus forward-facing child restraints: an updated assessment. Injury prevention, injuryprev-2017. 2018. 10.1136/injuryprev-2017-042512 29175832

[CR14] NHTSA. Child Restraint Use in 2008 – Use of Correct Restraint Types. 2009; https://crashstats.nhtsa.dot.gov/Api/Public/ViewPublication/811132. Accessed 18 May 2017.

[CR15] NHTSA. New Child Seat Guidelines. 2011; https://www.nhtsa.gov/equipment/car-seats-and-booster-seats. Accessed 18 May 2017.

[CR16] NHTSA (2015). Fatality Analysis Reporting System (FARS) Analytical User's Manual 1975-2014.

[CR17] NHTSA. Seat Belt Use in 2015 — Overall Results. 2016. https://crashstats.nhtsa.dot.gov/Api/Public/ViewPublication/812243. Accessed 18 May 2017.

[CR18] Oh Shin, Liu Chang, Pressley Joyce (2017). Fatal Pediatric Motor Vehicle Crashes on U.S. Native American Indian Lands Compared to Adjacent Non-Indian Lands: Restraint Use and Injury by Driver, Vehicle, Roadway and Crash Characteristics. International Journal of Environmental Research and Public Health.

[CR19] O'Neil J, Bandy R, Talty JL, Bull MJ (2011). Drivers’ reasons for choosing forward facing car safety seats. Clin Pediatr.

[CR20] SAS Institute Inc. In: SAS Institute Inc, editor. Base SAS® 9.4 Procedures Guide. Cary; 2014.

[CR21] Sauber-Schatz EK, West BA, Bergen G (2014). Vital signs: restraint use and motor vehicle occupant death rates among children aged 0–12 years—United States, 2002–2011. Morb Mortal Wkly Rep.

[CR22] Winston FK, Chen IG, Elliott MR, Arbogast KB, Durbin DR (2004). Recent trends in child restraint practices in the United States. Pediatrics.

